# Urticaria

**Published:** 1898-02

**Authors:** 


### Urticaria.

Dr. Bernard Wolff, of Atlanta, Ga., has become thoroughly convinced of the value of phosphate of soda in the treatment of urticaria. He has used this drug for the past year in a large number of cases, and with such uniform success that he regards it as absolute master of the disease.

In acute cases in adults he gives drachm doses of a supersaturated solution of phosphate of soda; that is, sixty to eighty grains to the drachm, the salt being dissolved in its own water of crystallization. This is repeated every three hours, and in addition the following antipruritic lotion is recommended:

Pulvis calamin and zinc oxid, aa ʒiss; acid. carbolic, ʒss; aq. calcis, ℥ij; aq. rosæ, ad ℥iv.

This is to be applied frequently and freely. The dose of the salt may be appropriately reduced in the cases of children, and the quantity of carbolic acid in the lotion diminished. The effect is prompt; within a few hours the acute symptoms subside, and in from twelve to twenty-four hours the eruption ceases to appear.

In the chronic type of the disease the relief afforded is equally prompt, but recurrences are apt to follow. The remedy should be given in doses of one drachm or more after meals and should be continued until all tendency to recurrence of the attacks has disappeared. Patients do not acquire a tolerance for the drug, and therefore it may be administered in unchanging doses as long as is necessary without any injurious effect.

The remedy is especially applicable in urticaria of gastro-intestinal origin, and is consequently applicable to fully ninety per cent of all cases.

Sodium phosphate is regarded as one of the most reliable hepatic stimulants and intestinal antiseptics, and its curative influence over urticaria is probably based upon this physiological action.—*Medical Standard.*

